# Resolution of Extensive Coronary Thrombosis under Rivaroxaban Treatment

**DOI:** 10.5935/abc.20150052

**Published:** 2015-12

**Authors:** Murat Yuksel, Abdulkadir Yildiz, Umit Tapan, Faruk Ertas, Sait Alan

**Affiliations:** 1 Dicle University, School of Medicine, Cardiology Department, Diyarbakir - Turkey; 2 Boston University Medical Center, Hematology/Oncology Department, Boston - USA

**Keywords:** Coronary Thrombosis / therapy, Anticoagulants, Blood Coagulation / genetics

## Introduction

Genetic mutations resulting in a hypercoagulable state are well-defined risk factors for venous thrombosis. However, there are few cases of arterial thrombosis, including coronary thrombosis, in patients with such genetic mutations described in the literature.

## Case Report

**Figure 1:**
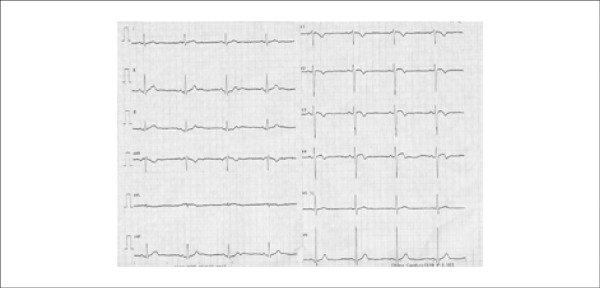
Electrocardiogram on admission showing biphasic T wave changes in leads V^2-4^.

**Figure 2:**
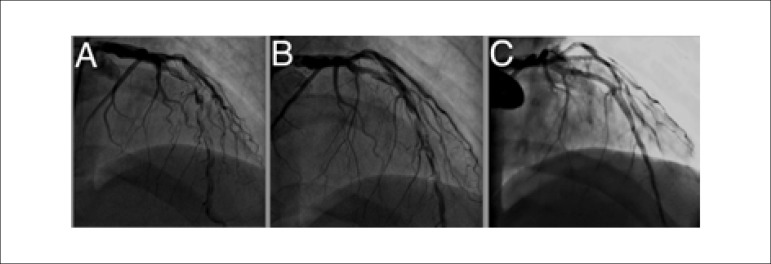
Antero-posterior cranial view showing left anterior descending artery **A**. At presentation with widespread thrombotic foci, **B**. After tirofiban infusion with a slight regression of thrombotic foci and C. Complete resolution of thrombotic foci after use of rivaroxaban for 8 weeks.

A 26-year-old male admitted to the emergency department with a new-onset chest pain for two days. His physical examination was unremarkable. Electrocardiogram (ECG) showed biphasic T wave changes in leads V_2-4_(Figure 1). The patient had a history of warfarin use for 6 months due to a deep vein thrombosis (DVT) suffered 2 years before. Bedside echocardiogram revealed slight hypokinesis of anterior wall with an ejection fraction of 52%. In laboratory analysis, creatinine kinase-MB and troponin-I levels were slightly elevated (32 ng/ml and 0.44 ng/ml, respectively). The patient was transferred to the coronary care unit with the diagnosis of non-ST segment elevation myocardial infarction. Coronary angiogram revealed multiple segmentary thrombotic foci along the left anterior descending (LAD) artery with Thrombolysis In Myocardial Infarction (TIMI)-III flow and normal circumflex and right coronary arteries (Figure 2A, Video 1). Manual thrombus aspiration or thrombectomy device were not considered because of the widespread nature of thrombosis throughout LAD. Initially, intracoronary tirofiban at a 10-µg/kg dose was given in 3 minutes, followed by 0.15 µg/kg/min intravenous infusion for 24 hours in addition to subcutaneous enoxaparin. Control angiogram revealed slight improvement (Figure 2B, Video 2). Then the patient was started on rivaroxaban 20 mg daily, for 8 weeks and control angiogram revealed complete resolution of the thrombus (Figure 2C, Videos 3 and 4). Meanwhile, thrombophilia work up resulted in prothrombin gene mutation (homozygous, G20210A) and homozygous mutation in plasminogen activator inhibitor type 1 (PAI-1) gene [4G/4G]. The patient also had a lupus anticoagulant at the time of initial presentation but did not have anti-cardiolipin antibodies or anti-beta2 glycoprotein-I antibodies. Also, his lupus anticoagulant was negative.

**Video 1:** Coronary angiogram showing the filling defects throughout the left anterior descending artery on antero-posterior cranial projection at presentation.

**Video 2:** A slight regressions of thrombotic foci is seen on antero-posterior cranial projection of angiogram after tirofiban infusion.

**Video 3:** Coronary angiograms demonstrating resolution of extensive thrombotic foci in left anterior descending artery on antero-posterior cranial projection after use of rivaroxaban for 8 weeks.

**Video 4:** Coronary angiograms demonstrating resolution of extensive thrombotic foci in left anterior descending artery on right cranial projection after use of rivaroxaban for 8 weeks.

## Discussion

The key clinical feature in this case is identifying the cause of the extensive coronary thrombosis in such a young man. We concentrated on thrombophilia because the patient had a history of DVT without any predisposing cause.

The plasminogen activator inhibitor-1 (PAI-1) level is crucial in the regulation of plasminogen activity and high levels of PAI-1 are associated with a pro-thrombotic state. A common guanosine insertion/deletion gene polymorphism of 4G/5G located at the 675^th^ base pair upstream of the start point of translation regulates PAI-1 levels. Homozygosity for the deletion genotype (4G/4G) has been shown to cause higher levels of PAI-1 compared to 4G/5G genotype^1^. The clinical significance of homozygous PAI-1 mutation by itself is not clear, but there are reports suggesting increased frequency of thrombotic events when found together with other pro-thrombotic situations^2,3^. The association between prothrombin gene mutation and thrombosis is well established; this mutation confers a 2.8 fold increase in the thrombotic risk.^4^ Our patient had a history of DVT 2 years prior to this presentation, suggesting his tendency to develop thrombosis. After obtaining the results of hypercoagulable work up, we felt imperative to treat him with an anticoagulant acutely as well as for lifelong. Rivaroxaban, a direct factor-Xa inhibitor approved for venous thromboembolism treatment/prevention, was chosen because of its easy administration compared to the subcutaneous and intravenous forms of heparin products and quick action and fewer drug interactions compared to warfarin.

This case stands as the first report of a patient presenting with acute coronary syndrome caused by widespread non-occlusive thrombotic foci in coronary artery due to prothrombin gene mutation and/or PAI-1 mutation. Additive effects of hypercoagulable states are well known and this case reiterates that fact with an unprovoked venous thrombosis and a widespread arterial thrombotic episode in a young patient showing no other known risk factors for arterial or venous thrombosis. It also highlights the importance of hypercoagulable work up in such patients.

## Conclusion

We demonstrated successful resolution of the widespread coronary thrombosis with rivaroxaban 20 mg daily, which was important to confirm the non-atherosclerotic nature of the occlusion.
